# Clinical manifestations in two patients with pyruvate dehydrogenase deficiency and long-term survival

**DOI:** 10.1038/hgv.2017.20

**Published:** 2017-06-01

**Authors:** Takanobu Yoshida, Jun Kido, Hiroshi Mitsubuchi, Shirou Matsumoto, Fumio Endo, Kimitoshi Nakamura

**Affiliations:** 1 Department of Pediatrics, Graduate School of Medical Sciences, Kumamoto University, Kumamoto, Japan

## Abstract

Pyruvate dehydrogenase E1-alpha deficiency (PDHAD) results in lactic acidosis and hyperpyruvatemia. Two patients with PDHAD, a man with a p.R263Q mutation, and a girl with a p.C145del mutation in *PDHE1α*, presented with lactic acidosis with neurological disorder. These patients were able to survive for a long period under careful nursing care. Herein, we discuss the factors contributing to their relatively stable clinical course, albeit with intellectual disability.

Pyruvate dehydrogenase (PDH) catalyzes the irreversible decarboxylation of pyruvate into acetyl-CoA, linking glycolysis to the tricarboxylic acid cycle. PDH is composed of multiple copies of three subunits, that is, PDH (E1, EC 1.2.4.1), dihydrolipoamide transacetylase (E2, EC 2.3.1.12) and dihydrolipoamide dehydrogenase (E3, EC 1.8.1.4), along with an E3-binding protein. The E1 component is a heterotetramer of two α-subunits and two β-subunits. The gene encoding the E1α subunit (*PDHA1*, MIM #300502) is located on the X chromosome (Xp22.1–22.2), and all subunits are nuclear-encoded. PDH E1α deficiency (PDHAD, MIM #312170) results in lactic acidosis and hyperpyruvatemia. The spectrum of the clinical manifestations of PDHAD ranges from severe neonatal lactic acidosis with early death to intermittent ataxia with a late-onset progressive neurodegenerative course.^1,2^

We followed two PDHAD patients with intellectual disability and a relatively stable clinical course under careful nursing care. We previously reported the onset and diagnosis of patient 1,^3,4^ whereas patient 2 presents a novel mutation (c.433_435delTGT, p.C145del mutation; http://www.hgmd.cf.ac.uk). We report the relationship between their genetic backgrounds and long-term outcomes.

Patient 1 is a 38-year-old man, who had been born at full term with a birth weight of 2,670 g. He could support his head at 6 months and could stand with support at 14 months. He first presented with apnea at 19 months and was referred to our institution. He presented with severe metabolic acidosis (blood pH, 7.30; HCO_3_^−^, 7.7 mEq/l; base excess, −15.3 mEq/l), hyperlactacidemia (50.8 mg/dl) and hyperpyruvic acidemia (3.4 mg/dl). Lactate (31 mg/dl) and pyruvate (2.1 mg/dl) levels were elevated in his cerebrospinal fluid (CSF). Brain CT revealed microcephaly and a low-density area on both sides of the basal ganglia. At 21 months, he was diagnosed with a c.G788A *de novo* mutation in *PDHA1* exon 8 (NM_000284.3), resulting in an amino acid substitution (p.R263Q). He was treated with vitamins B1 and B6. He was repeatedly hospitalized (two to four times per year) during his childhood because of convulsions and infections. His body condition gradually deteriorated at every hospitalization; he could not walk and presented with repeated aspiration pneumonia. He underwent gastrostomy at 28 years of age and laryngo-tracheal resection at 29 years of age. Thereafter, he had fewer convulsions and was never hospitalized again because further pneumonia was prevented. He lives at home with his guardian without respiratory support.

Patient 2 is a 10-year-old girl, the first child of nonconsanguineous parents. She presented with intrauterine growth restriction at 9 months of gestation. She was delivered by urgent caesarean section at 35 weeks of gestation because of preeclampsia. Her birth weight was 1,716 g. She presented mild metabolic acidosis (blood pH, 7.36; HCO_3_^−^, 18.0 mEq/l; base excess, −6.2 mEq/l). Head ultrasonography indicated ventricular enlargement on both sides and periventricular leukomalacia (Figure 1a,b). Her brain MRI at 31 days of age showed symmetrical low-intensity signals of the basal ganglia and corpus callosum hypoplasia (Figure 1c). PDHAD was suspected because of elevated blood lactate (64.7 mg/dl) and pyruvate (5.0 mg/dl) and lactate in the CSF (58.4 mg/dl) (Supplementary Table S1). She was diagnosed with a c.433_435delTGT heterozygous mutation in *PDHA1* exon 5 (NM_000284.3) (Figure 1d–f), confirmed to be *de novo*. She was treated with vitamin B1, biotin, lipoate and dichloroacetic acid (DCA). Although hyperlactacidemia and hyperpyruvic acidemia improved to 18.4 mg/dl and 1.78 mg/dl, respectively, lactate levels in the CSF (44.1 mg/dl) had not decreased 12 days after administration. As the ventricular enlargement and brain atrophy progressed with time, DCA was discontinued at 3 years of age. She was hospitalized three times because of decreased appetite, convulsions and aspiration pneumonia. She underwent cardioplasty at 4 years of age because her gastroesophageal reflux became severe. She then demonstrated good nutritional status and did not develop aspiration pneumonia again. She is now 10 years old and has a developmental quotient of <20 on the Enjoji scale of infant analytical development and an unmeasurable intelligence quotient. She is in a stable physical state and goes to a special school.

This case report was approved by the Ethics Committee of the Faculty of Life Science, Kumamoto University. Written informed consent was obtained from the families of both patients reported herein.

No report has been published regarding patients with PDHAD who have survived as long as patient 1. Although patient 2 has fetal-onset type PDHAD, she presents with a relatively stable health condition. Patel *et al.*^5^ reported that 243 of 371 patients with PDHAD (65.5%) were alive at 6 months of age, but only 10 patients (2.7%) were still alive at 20 years of age. Death was associated with high blood lactate levels and low PDH activity. The common *PDHA1* mutations are at amino acid position 263, 302 or 378. Some patients with c.R263G survive for 7–18 years.^6,7, 8^ Patient 1 presented with p.R263Q and has survived for 38 years to date. The enzyme activity is variable between male patients with p.R263G and p.R263Q (16–77%; Table 1). According to Barnerias *et al.*^9^ and DeBrosse *et al.*,^10^ within specimens from the same individual with *PDHA1* mutations and even within the same specimen, there are large variations in PDH activity. This was evident when PDH activity was compared between cultured skin fibroblasts and fresh blood lymphocytes from the same individual. DeBrosse *et al.*^10^ reported a significant difference in the survival of males with *PDHA1* mutations whose fibroblast PDH activity was ⩾35% of the reference mean compared with those whose PDH activity was <35% of the mean. However, there was no significant correlation between PDH activity in fibroblasts from male or female patients with *PDHA1* mutations and their intellectual outcome. Therefore, male subjects with *PDHA1* mutations whose fibroblast PDH activity is ⩾35% are more likely to have long-term survival, similar to patient 1 reported here.

The c.433_435delTGT (p.C145del) identified in patient 2 has never been reported. Gene mutations around amino acid 145 in exon 5 are summarized in Table 1. Patients with p.R141Q or p.G144del present residual activity, and the activity of p.C145del might be relatively high, although this information is not available. However, investigating PDH activity remains difficult, particularly in female patients with *PDHA1* mutations, because of tissue-dependent X-inactivation. The defect might need to be sought and confirmed in several tissues by techniques such as enzymatic activity assay and immnohistochemistry.^11,12, 13,^ Tissue-specific skewed X-chromosome inactivation gives rise to the variability of female PDHAD.

The effective treatment for PDHAD is a ketogenic diet with carbohydrate restriction. Patients 1 and 2 consumed a ketogenic diet. Biotin was administered to both patients to activate their pyruvate carboxylase, which catalyzes pyruvate carboxylation to form oxaloacetate. Moreover, vitamin B1 and DCA might be useful. DCA was only administered to patient 2. DCA decreases lactate and pyruvate levels in the blood and CSF by inhibiting PDH kinase and stabilizing the PDH complex. After DCA administration in patient 2, lactate levels decreased in the blood, but not in the CSF at 12 days after administration. Neurological symptoms gradually improve from 1 to 24 months after administration of DCA.^14^ Thus, improvement in CSF and neurological symptoms might be observable after at least a month of DCA administration.

Although patient 1 achieved head control late, he underwent a detailed examination for PDHAD as late as at 19 months. Intervention at an earlier stage might have improved the outcome. He was repeatedly hospitalized during his childhood because of convulsions and infections, and his general physical condition deteriorated every time he contracted an infection. Moreover, his general medical condition deteriorated further after receiving dextrose infusion.

In patient 2, ventricular enlargement was recognized at 9 months of pregnancy. Ultrasonography and MRI demonstrate brain dysgenesis in fetuses with PDHAD.^15^ Brain function depends on the energy produced from aerobic glucose oxidation. Thus, PDHAD negatively affects brain development. PDHAD should be considered as a differential diagnosis when abnormal brain structure is detected in fetuses. Moreover, when PDHAD is definitely diagnosed, we consider that it is important to administer a ketone formula containing low carbohydrate content and sufficient vitamin B1. Vitamin C, Vitamin E, biotin, coenzyme Q10 and carnitine might also help mitochondrial function because the two patients in this case report had a clinical course without significant change when receiving these supplements; however, the use of these supplements for PDHAD continues to be controversial.^16,17,18,19^

In conclusion, early and long-term interventions should be tailored according to each patient’s symptoms and problems. Good nutritional conditions should be maintained, while controlling convulsions and preventing aspiration pneumonia.

## Figures and Tables

**Figure 1:**
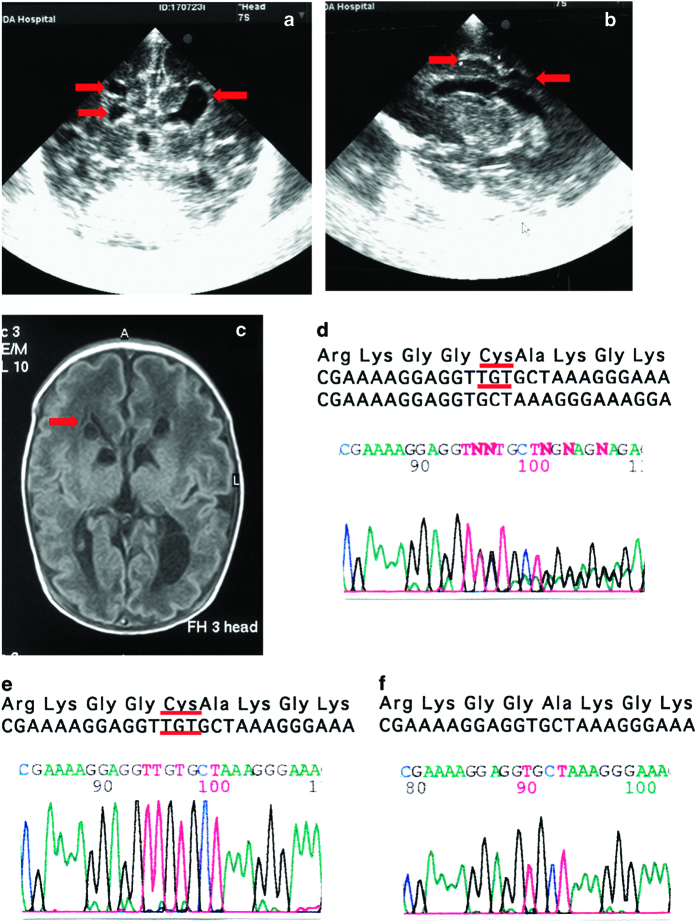
Brain imaging and *PDHA1* DNA sequence in patient 2. Brain ultrasonography ((**a**) coronal section and (**b**) sagittal section images) demonstrated enlarged lateral ventricles on both sides and cystic lesions. (**c**) Brain MRI showed enlarged lateral ventricles on both sides and cystic changes around the lateral ventricles. (**d**) Direct *PDHA1* DNA sequencing of white blood cell DNA in patient 2. Subcloning of PCR products generated from white blood cell DNA from patient 2 demonstrated a mixture of the normal sequence (**e**) and the c.433_435delTGT (p.C145del) mutation (**f**).

**Table 1:** *PDHA1* mutations in patients 1 and 2 and in the literature

Exon	Nucleotide change (amino acid change)	Sex	Mother	PDHc activity (%)	Phenotype	Reference
8	c.788G>A (p.R263Q)	M	NC	38	Leigh	Case 1
8	c.787C>G (p.R263G)	F	C	53	Dystonia	U(KDS)^6^
8	c.787C>G (p.R263G)	M	NC	50	Lactic acidosis and ataxia	U(KDS)^6^
8	c.787C>G (p.R263G)	M	NA	30	Lactic acidosis and ataxia	Wexler *et al.*^20^
8	c.787C>G (p.R263G)	M	C	77	Lactic acidosis and mental retardation	Wexler *et al.*^21^
8	c.787C>G (p.R263G)	M	NC	16	Mental retardation and ataxia	Chun *et al.*^8^
8	c.787C>G (p.R263G)	M	C	49	Mental retardation and ataxia	Chun *et al.*^8^
8	c.787C>G (p.R263G)	M	C	39	Mental retardation and ataxia	Chun *et al.*^22^
8	N.A (p.R263G)	M	C	50	Leigh	Briones *et al.*^23^
8	c.787C>G (p.R263G)	M	C	31	Leigh	Lissens *et al.*^24^
8	N.A (p.R263G)	M	NC	46	Leigh	Marsac *et al.*^25^
8	N.A (p.R263G)	M	NC	NA	Leigh	Marsac *et al.*^25^
8	c.787C>G (p.R263G)	M	C	NA	Leigh	Naito *et al.*^26^
5	c.433_435delTGT (p.C145del)	F	NC	NA	Neonatal lactic acidosis, necrosis of basal ganglia and periventricular leukomalacia	Case 2
5	c.422G>A (p.R141Q)	M	NA	32	Neonatal lactic acidosis and corpus callosum agenesis	U(BGK)^6^
5	c.429_431delAGG (p.G144del)	M	NA	16	Lactic acidosis	Ostergaard *et al.*^27^

Abbreviations: C, carrier; F, female; M, male; NA, not available; NC, not a carrier.

U(BGK,KDS) refers to unpublished patients with mutations identified by the research teams of Brown and colleagues or Kerr and colleagues.

PDH complex (PDHc) activity was measured in patient fibroblasts.
