# Supplementary material for: Clinical manifestations in two patients with pyruvate dehydrogenase deficiency and long-term survival
Source: Hum Genome Var. 2017 Jun 1;4:17020–. doi: 10.1038/hgv.2017.20 (PMC5451473; doi:10.1038/hgv.2017.20)
Supplement: Supplementary Table [file hgv201720-s1.docx]

Supplemental Table: Clinical data of Case 2 on admission to our institute.

| Blood biochemistry |  | Blood  coagulation test |  |
| --- | --- | --- | --- |
| TP | 4.2 (mg/dL) | PT | 77 (%) |
| Alb | 3.6 (mg/dL) | APTT | 53 (%) |
| T-Bil | 10.3 (mg/dL) | HPT | 49 (%) |
| T-BA | 10.3 (IU/L) | Fib | 223 (mg/dL) |
| AST | 13 (IU/L) | AT III | 48 (%) |
| ALT | 12 (IU/L) | Venous blood gas |  |
| γGTP | 112(IU/L) | pH | 7.361 |
| LDH | 200 (IU/L) | pCO2 | 32.5 (mmHg) |
| CHE | 159 (IU/L) | HCO_3_^-^ | 18.0 (mmol/L) |
| BUN | 1.9 (mg/dL) | BE | -6.2 (mmol/L) |
| Cre | 0.28 (mg/dL) | Lac | 7.6 (mmol/L) |
| CK | 77 (IU/L) |  |  |
| CRP | <0.01 (mg/dL) |  |  |
| BS | 84 (mg/dL) |  |  |
| Na | 141 (mEq/L) |  |  |
| K | 4.7 (mEq/L) |  |  |
| Cl | 107 (mEq/L) |  |  |
| Ca | 10.3 (mg/dL) |  |  |
| UA | 2.2 (mg/dL) |  |  |
| Fe | 68 (mg/dL) |  |  |
| UIBC | 103 (µg/dL) |  |  |
| NH_3_ | 72 (µmol/L) |  |  |
| LA | 64.7 (mg/dL) |  |  |
| PA | 5.0 (mg/dL) |  |  |
| L/P | 12.9 |  |  |
| CBC |  |  |  |
| WBC | 5700 (/µL) |  |  |
| Hb | 9.4 (g/dL) |  |  |
| Plt | 28.6 × 10^4^ (/μL) |  |  |

TP: total protein, Alb: Albumin, T-Bil: total bilirubin, T-BA: AST: aspartate aminotransferase, ALT: alanine aminotransferase, γGTP: [γ glutamyl transpeptidase](http://ejje.weblio.jp/content/%CE%B3%E2%80%90glutamyl+transpeptidase), LDH: [lactase dehydrogenase](javascript:void(0);), CHE: choline esterase, BUN: blood urea nitrogen, Cre: creatinine, CK: Creatine kinase, CRP: [C-reactive protein](javascript:void(0);), BS: Blood sugar, UA: Uric acid, UIBC: Unsaturated iron binding capacity, LA: Lactic acid, PA: **Pyruvic acid,** CBC: [complete blood](javascript:void(0);) [cell](javascript:void(0);) [count](javascript:void(0);), WBC: white blood cell count, Hb: hemoglobin, Plt: platelets, PT: prothrombin time, APTT: activated partial thromboplastin time, Fib: fibrinogen, AT III: antithrombin III, BE: Base excess
